# Hypotensive Response to Angiotensin II Type 2 Receptor Stimulation in the Rostral Ventrolateral Medulla Requires Functional GABA-A Receptors

**DOI:** 10.3389/fnins.2017.00346

**Published:** 2017-06-19

**Authors:** Laura Légat, Sofie Brouwers, Ilse J. Smolders, Alain G. Dupont

**Affiliations:** ^1^Laboratory of Pharmaceutical Chemistry, Drug Analysis and Drug Information (FASC), Research Group Experimental Pharmacology (EFAR), Center for Neurosciences (C4N), Vrije Universiteit BrusselBrussels, Belgium; ^2^Cardiovascular Center, Universitair Ziekenhuis BrusselBrussels, Belgium; ^3^Department of Clinical Pharmacology and Clinical Pharmacy, Universitair Ziekenhuis BrusselBrussels, Belgium

**Keywords:** renin-angiotensin-system, angiotensin II type 2 receptor, compound 21, mean arterial pressure, gamma-aminobutyric acid, rostral ventrolateral medulla

## Abstract

**Objectives:** Angiotensin II, glutamate and gamma-aminobutyric acid (GABA) interact within the rostral ventrolateral medulla (RVLM) and the paraventricular nucleus (PVN) modulating the central regulation of blood pressure and sympathetic tone. Our aim was to assess the effects of local angiotensin II type 2 receptor stimulation within the RVLM and the PVN on neurotransmitter concentrations and mean arterial pressure (MAP).

**Methods:** *In vivo* microdialysis was used for measurement of extracellular glutamate and GABA levels and for local infusion of the angiotensin II type 2 receptor agonist Compound 21 in the RVLM and the PVN of conscious normotensive Wistar rats. The MAP response to local Compound 21 was monitored with a pressure transducer under anaesthesia. Angiotensin II type 2 receptor selectivity was assessed using the angiotensin II type 2 receptor antagonist PD123319; the GABA-A receptor antagonist bicuculline was used to assess the involvement of GABA-A receptors.

**Results:** Infusion of Compound 21 (0.05 μg/μl/h) in the RVLM significantly increased GABA levels and lowered blood pressure. These effects were abolished by co-infusion with PD123319. No changes in neurotransmitter levels or effects on blood pressure were seen with PD123319 infusion alone. Co-infusion of bicuculline abolished the Compound 21 evoked decrease in MAP. Infusion of Compound 21 within the PVN did not change extracellular neurotransmitter levels nor MAP.

**Conclusion:** Selective stimulation of angiotensin II type 2 receptor within the RVLM by local Compound 21 infusion reduces blood pressure and increases local GABA levels in normotensive rats. This hypotensive response requires functional GABA-A receptors, suggesting that GABAergic neurons are involved in the sympatho-inhibitory action underlying this hypotensive response.

## Introduction

Angiotensin II (Ang II) is the most important effector within the renin-angiotensin-aldosterone system (RAAS), mediating its actions through the angiotensin II type 1 receptors (AT1R), and angiotensin II type 2 receptors (AT2R). Activation of the AT1R mediates most known effects of Ang II, such as vasoconstriction, renal sodium retention, promotion of inflammatory responses, vascular smooth muscle cell proliferation and hypertrophy. The AT2R counteracts these AT1R effects and mediates vasodilation, apoptosis, natriuresis and anti-inflammatory, anti-proliferative and anti-fibrotic responses (de Gasparo et al., 2000; Padia and Carey, 2013). It has been demonstrated that activation of the AT2R, part of the “protective arm” of the RAAS, leads to therapeutic protective effects against myocardial and brain injury (Namsolleck et al., 2014). Despite the fact that AT2R stimulation causes vasodilation *ex* and *in vivo*, due to the dominating AT1R mediated vasoconstrictor tone, peripheral AT2R stimulation *in vivo* does not cause lowering in blood pressure (Steckelings et al., 2012).

The brain RAAS plays a major role in the regulation of blood pressure and sympathetic tone and that brain Ang II induces tonic sympatho-excitatory effects resulting in blood pressure increases through stimulation of central AT1R (Guyenet, 2006; Dupont and Brouwers, 2010). However, the possible role of the central AT2R herein are incompletely understood although recent data support the involvement of central AT2Rs in the regulation of blood pressure and sympathetic tone (Gao and Zucker, 2011; Li et al., 2012). Intracerebroventricular (icv) injection of Ang II in AT2R-knockout mice was reported to result in a larger increase in blood pressure compared to wild type mice, suggesting a counter-regulatory protective role of brain AT2R in the regulation of blood pressure (Siragy et al., 1999; Li et al., 2003).

The development of the first orally active, selective, non-peptide agonist of the AT2R, Compound 21 (C21) offers the possibility to selectively and specifically investigate AT2R mediated effects (Wan et al., 2004; Steckelings et al., 2011). C21 was reported to induce cardio-, cerebro-, and nephroprotective as well as anti-inflammatory effects in different animal models. Although we, as others, could not demonstrate a putative hypotensive response after peripheral administration of a range of different doses of C21, with or without concomitant AT1R blockade (Yang et al., 2011; Brouwers et al., 2013, 2015), we did observe significant blood pressure decreases after chronic icv infusion of C21 (Yang et al., 2011; Brouwers et al., 2015). Specific and selective stimulation of brain AT2R with C21 evoked a sustained hypotensive response not only in normotensive but also in spontaneously hypertensive rats *in vivo*. In addition, we observed that this hypotensive response was associated with sympatho-inhibition and increased spontaneous baroreflex sensitivity (Steckelings et al., 2012; Brouwers et al., 2015).

The central regulation of blood pressure involves different parts of the brain. However, the most important site within the brainstem, involved in the short- and longterm central regulation of the blood pressure is the rostral ventrolateral medulla (RVLM) region, the so-called “pressor area,” which is responsible for the sympathetic drive. The RVLM receives inputs from multiple integrative areas in the hypothalamus and the medulla and is the main region from which the sympathetic outflow from the brain originates (Guyenet, 2006; Dupont and Brouwers, 2010). The neurons in the paraventricular nucleus (PVN) of the hypothalamus have projections to the RVLM and are also known to significantly affect sympathetic output indirectly through modulation of the neurons within the RVLM region (Guyenet, 2006; Dupont and Brouwers, 2010). Therefore, the RVLM and the PVN are generally considered the two most appropriate sites to study the central regulation of sympathetic activity. Neuronal excitability in the RVLM and the PVN are mainly modulated by the “classical” excitatory and inhibitory neurotransmitters, glutamate and gamma-aminobutyric acid (GABA), respectively (Miyawaki et al., 1996; Butcher and Cechetto, 1998; Tasker et al., 1998; Li et al., 2006; Hatam and Ganjkhani, 2012).

Brain Ang II, acting through AT1R, increases the sympathetic outflow through stimulation of glutamatergic neurons in the RVLM (Dupont and Brouwers, 2010). The presence of AT2R in the RVLM opposing the effect of neuronal stimulation through the AT1R has also been demonstrated (Gao et al., 2008a). Current evidence suggests that AT2R in the RVLM may mediate a sympatho-inhibitory effect (Gao et al., 2008a,b). Brain angiotensin peptides, glutamate and GABA appear to interact within the RVLM and the PVN to regulate sympathetic tone and blood pressure (Li et al., 2006; Dupont and Brouwers, 2010).

In the present study we aimed to further investigate the possible role of AT2R located within the RVLM-PVN axis and their interaction with glutamate and GABA in the central regulation of blood pressure. We therefore assessed blood pressure changes and possible effects on local glutamate and GABA concentrations in response to local unilateral administration of C21 within the RVLM and the PVN through microdialysis.

## Materials and methods

### Animals

All experiments were carried out on normotensive male albino Wistar rats (Charles River Laboratories, France) weighing between 250 and 300 g. Animals were kept in the animal house of the Vrije Universiteit Brussel minimum 1 week before surgery at constant temperature (24°C) and relative humidity (50%) with 12 h light-dark cycle and had *ad libitum* food and water. All protocols used and described for animal experiments on rats (*n* = 4–9 per experimental group) were carried out according to the National and European guidelines for animal experimental research and were approved by the Ethical Committee for Animal Experiments of the Faculty of Medicine and Pharmacy of the VUB. All possible steps were taken to avoid animals' suffering at each stage of the experiment.

### Drugs

S-(+)-1-[(4-(Dimethylamino)-3-methylphenyl)methyl]-5-(diphenylacetyl)-4,5,6,7-tetrahydro-1H-imidazo[4,5-c]pyridine-6-carboxylic acid di(trifluoroacetate) salt hydrate (PD123319) and bicuculline were purchased form Sigma-Aldrich Co. (St. Louis, USA). Compound 21 (C21) was provided by Vicore Pharma AB (Göteborg, Sweden). Doses of C21, PD133319 and bicuculline were selected based on previous studies (Smolders et al., 1995a; Brouwers et al., 2015).

### Experimental protocol

Normotensive Wistar rats were first subjected to brain surgery, as described in “*surgical procedures*” for implantation of a guide cannula. The following day, samples were collected through *in vivo* microdialysis on freely moving rats allowing us to measure neurotransmitters at many time points in each animal. All dialysis samples were analyzed by high performance liquid chromatography for measurement of glutamate and GABA levels. On day three, the same rats as used for microdialysis experiments were anaesthetized in order to cannulate the right carotid artery for continuous monitoring of mean arterial pressure (MAP) with a pressure transducer and the pharmacological experiments were repeated.

Post-mortem evaluation was done after each experiment in order to exclude animals with inaccurately implanted probes.

### Surgical procedures

Animals were anaesthetized prior to surgery with a ketamine/diazepam mixture (90/4.5 mg/kg) intraperitoneally and received ketoprofen (4 mg/kg) subcutaneously. A stainless steel cannula (CMA12, Solna, Sweden) was stereotaxically implanted into the left RVLM (AP: −2.2, L: −12.3, V: 7) or left PVN (AP: −0.5, L: −1.8, V: 7), according to the atlas of Paxinos and Watson (1998) and fixed with dental cement.

### *In vivo* microdialysis

After overnight recovery, rats were single housed in experimental cages and the microdialysis probe (CMA12/1 mm membrane length, Solna, Zweden) was inserted into the guide-cannula. For collection of basal brain dialysates, the microdialysis probe was continuously perfused at a flow rate of 2 μl/min with modified Ringer's solution (147 mM NaCl, 2.3 mM CaCl_2_, 4 mM KCl). Samples were collected with a temporal resolution of 20 min and split into two aliquots of 15 μl. The experiment proceeded through perfusion with modified Ringer's solution for 120 min. Consecutively, one of the treatments at a rate of 2 μl/min: [C21 (0.05 μg/μl/h), PD123319 (0.05 μg/μl/h) or C21 (0.05 μg/μl/h) + PD123319 (0.05 μg/μl/h)] were dissolved in modified Ringer's solution and perfused through the microdialysis probe for 120 min and finally modified Ringer's solution for 120 min. Samples were stored at −20°C and thawed prior to analysis.

### Determination of glutamate and GABA dialysate levels via liquid chromatography

Two distinct chromatographic systems were used in the present study to determine glutamate and GABA dialysate levels following there derivatization with o-phtalaldehyde and β-mercapto-ethanol or tert-butylthiol respectively. Glutamate concentrations were determined using reversed-phase narrowbore liquid chromatography with gradient elution and fluorescence detection as described previously (Smolders et al., 1995b), while GABA was analyzed by reversed-phase microbore liquid chromatography with isocratic elution and electrochemical detection as described in detail in Van Hemelrijck et al. (2005).

### Mean arterial pressure (MAP) measurements

Animals were anaesthetized prior to surgery by 4% sevoflurane gas, and during surgery anaesthesia was maintained by 2.5% sevoflurane administration. The right jugular vein was catheterized for fluid maintenance (saline 0.9%), and the right carotid artery was cannulated for continuous monitoring of MAP with a pressure transducer (HP Hewlett Packard, Boebingen, Germany). The experimental protocol started after a 30 min equilibration period following surgery in order to record baseline values before the administration of the pharmacological compounds. The experiment proceeded through perfusion of modified Ringer's solution for 30 min. Consecutively, one of the treatments at a rate of 2 μl/min: [C21 (0.05 μg/μl/h), PD123319 (0.05 μg/μl/h), bicuculline (100 μM), C21 (0.05 μg/μl/h) + PD123319 (0.05 μg/μl/h) or C21 (0.05 μg/μl/h) + bicuculline (100 μM)] were dissolved in modified Ringer's solution and perfused through the microdialysis probe for 120 min, and finally modified Ringer's solution for 30 min.

### Post-mortem evaluation

At the end of every experiment, rats were killed by an overdose of Nembutal. In order to fix the brain, perfusing was performed by 4% paraformaldehyde solution and removed brains were preserved on formol. After slicing brain tissue, probe localization and tissue damage were histologically verified and evaluated post-mortem by a neutral red staining in order to exclude animals with inaccurately implanted probes.

### Statistical analysis

Data are expressed as mean ± standard error of the mean (SEM), all calculations and graphs were obtained using Graphpad Prism 4.03 (Graphpad Software Inc., San Diego, CA, USA). The mean values of the basal microdialysis samples obtained before drug administration were considered as the 100% baseline value for each animal. All neurotransmitter (glutamate and GABA) results were expressed as percentages of this baseline value ± SEM. All MAP measurements are shown as the MAP by SEM. For determination of intragroup differences of the treatment an one-way ANOVA for repeated measures followed by *post-hoc* Dunnett's multiple comparison test was used. Subsequently, for the microdialysis results, an area under the curve (AUC) analysis was performed to determine if there was an overall difference in neurotransmitter (glutamate and GABA) concentrations between different compounds. AUC values, expressed in arbitrary units, were compared by a Kruskal Wallis test with Dunn's multiple comparison *post-hoc* test. A value of *p* < 0.05 was considered to be statistically significant.

## Results

### AT2R-mediated changes in neurotransmitter concentrations in the RVLM

**Figure 1:**
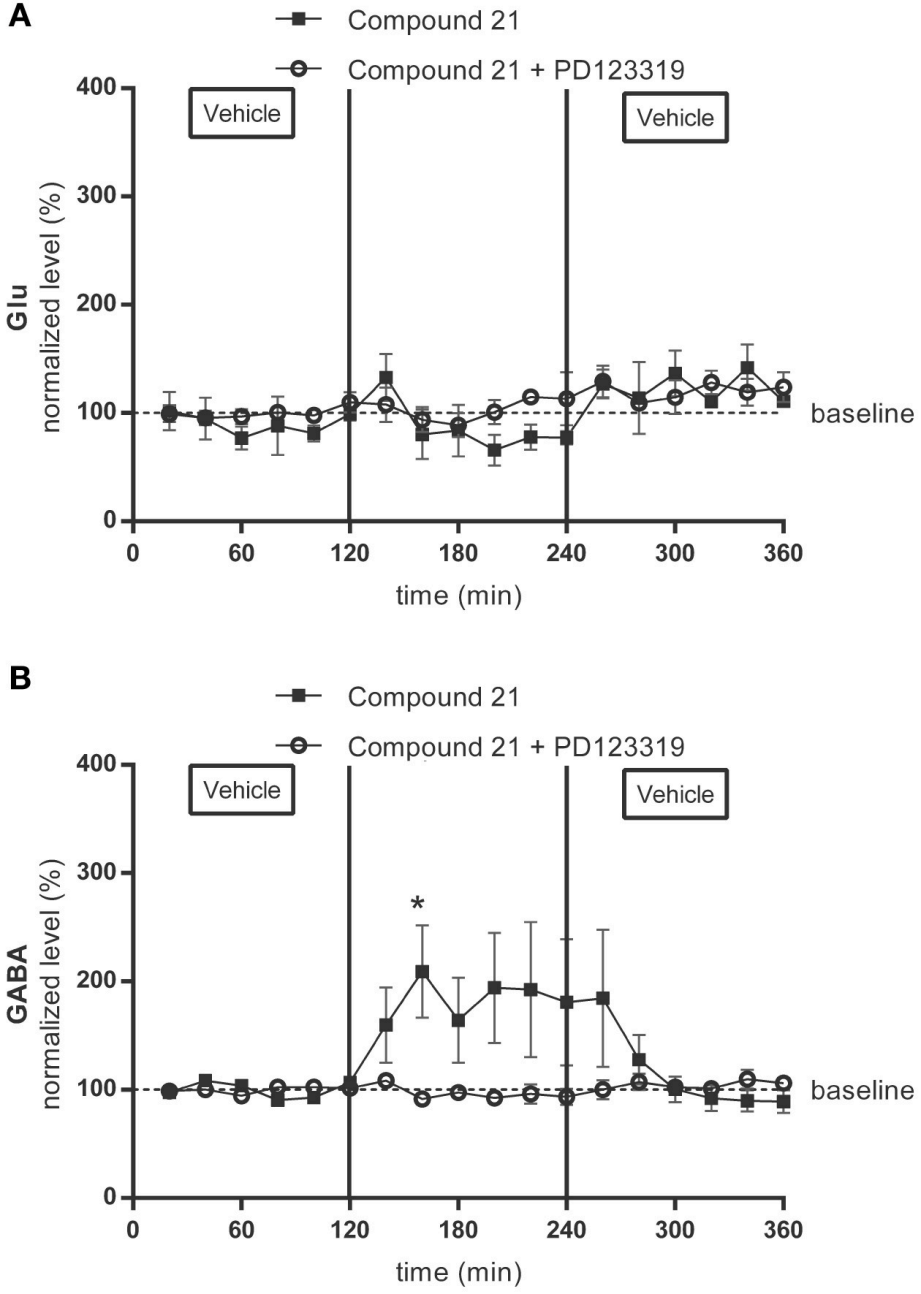
Microdialysis experiment: effect of AT2R stimulation and infusion of the AT2R agonist C21 alone (0.05 μg/μl/h) on the extracellular glutamate (Glu) **(A)** (*n* = 4) and GABA **(B)** (*n* = 6) concentrations and of co-infusion of C21 with the AT2R antagonist PD123319 (0.05 μg/μl/h) on the extracellular glutamate (Glu) **(A)** (*n* = 4) and GABA **(B)** (*n* = 5) concentrations in the RVLM in normotensive freely moving Wistar rats. Dialysates were collected every 20 min. C21 and C21 + PD123319 were dissolved in modified Ringer's solution and administered through the dialysis probe from time 120 to 240 min. Data are presented as the mean percentage of the baseline values (vehicle) ± SEM. Statistical analysis for intragroup differences of the treatment is performed using one-way ANOVA for repeated measures and the Dunnett's multiple comparison test; significant data compared to basal levels are indicated by asterisks (^*^p < 0.05).

The baseline values for glutamate and GABA in the microdialysis samples of the RVLM were 432 ± 219 nM for glutamate and 10 ± 5 nM for GABA in the group receiving 0.01 μg/μl/h C21 (data not shown), and 44 ± 12 nM for glutamate (Figure 1A) (*n* = 4) and 3 ± 1 nM for GABA (Figure 1B) (*n* = 6) in the group receiving 0.05 μg/μl/h C21. No changes from baseline levels were seen for glutamate or GABA levels during infusion of C21 (0.01 μg/μl/h) within the RVLM (data not shown). Infusion of the higher dose of C21 (0.05 μg/μl/h) within RVLM significantly increased GABA levels (*p* < 0.05; Figure 1B) but tended to decrease glutamate levels (Figure 1A), with a subsequent return to baseline levels for both transmitters after switching the infusion again to Ringer's solution alone.

In the group receiving 0.05 μg/μl/h PD123319 alone, average baseline dialysate concentrations of the RVLM were 354 ± 9 nM for glutamate and 8 ± 6 nM for GABA (data not shown). These values were not significantly different from the mean baseline levels during infusion with the AT2R antagonist.

In the group receiving 0.05 μg/μl/h C21 with PD123319, baseline concentrations were 113 ± 17 nM for glutamate (Figure 1A) (*n* = 4) and 14 ± 1 nM for GABA (Figure 1B) (*n* = 5). Neurotransmitter concentrations during co-infusion of C21 + PD123319 were not significantly different from the mean baseline levels. Co- infusion of the AT2R antagonist, PD123319 (0.05 μg/μl/h) with C21 (0.05 μg/μl/h) thus abolished the C21- evoked increase in GABA concentrations (Figure 1B).

**Figure 2:**
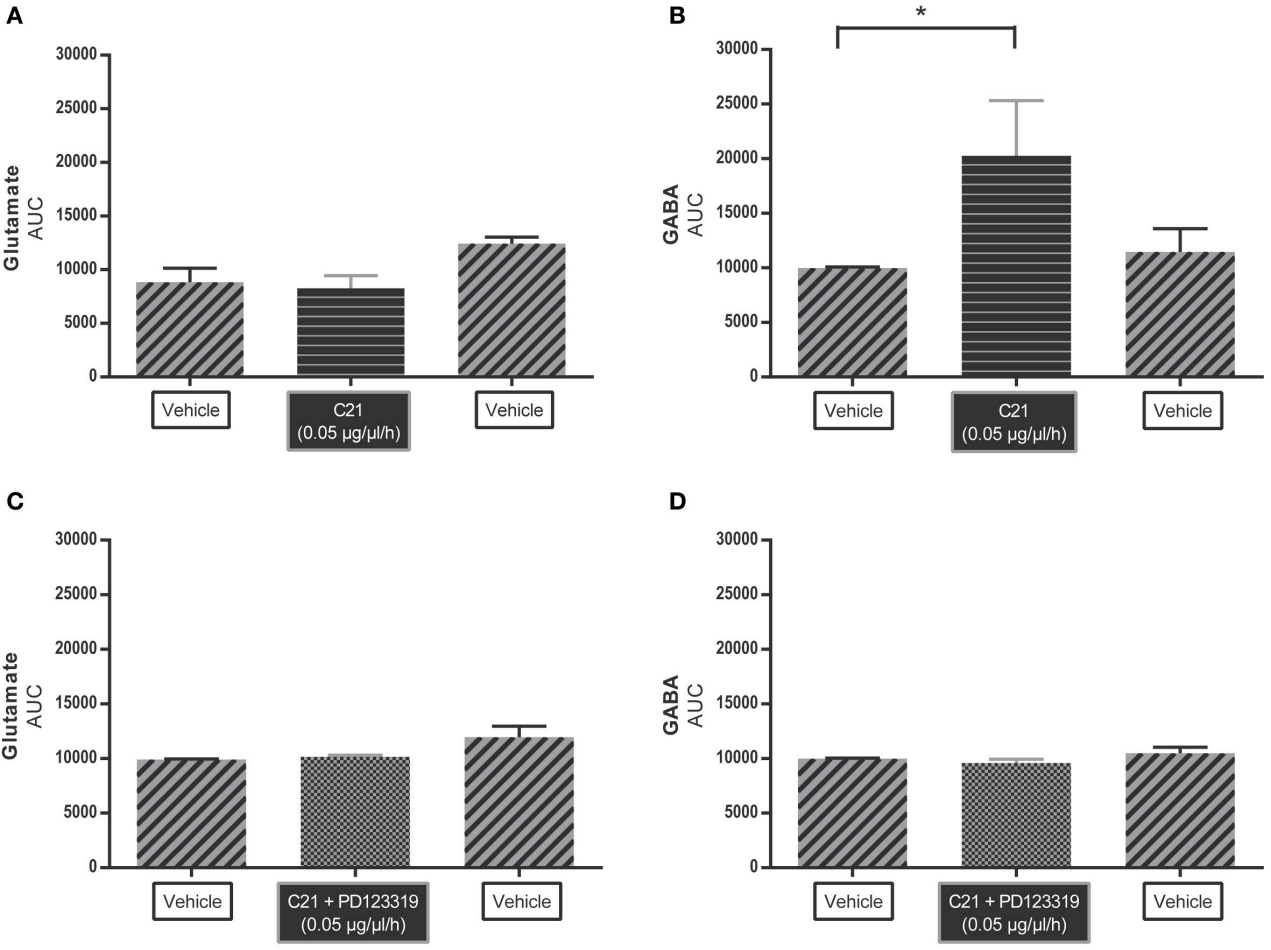
Microdialysis experiment: area under the curve (AUC) values of C21 alone **(A)** (*n* = 4) and **(B)** (*n* = 6) and in co-infusion with PD123319 **(C)** (*n* = 4) and **(D)** (*n* = 5) applied by microdialysis into the RVLM in normotensive freely moving Wistar rats on extracellular levels of glutamate **(A,C)** and GABA **(B,D)**. Data are represented as the mean AUC ± SEM. Statistical analysis for overall differences in neurotransmitter (glutamate and GABA) concentrations between different compounds is performed using Kruskal Wallis test with Dunn's multiple comparison test; significant data are indicated by asterisks (^*^p < 0.05).

Figure 2 shows the AUC values of glutamate and GABA dialysate levels under C21 infusion compared to vehicle infusion. AUC of glutamate levels (Figure 2A) (*n* = 4) did not significantly change under C21 (0.05 μg/μl/h) infusion or under C21 + PD123319 (0.05 μg/μl/h) infusion (Figure 2C) (*n* = 4). However, GABA levels (Figure 2B) (*n* = 6) significantly (*p* < 0.05) increased under C21 (0.05 μg/μl/h) infusion compared to baseline values. This increase in GABA levels mediated by C21 infusion was abolished by co-infusion with PD123319 (Figure 2D) (*n* = 5).

### Lack of AT2R-mediated changes on neurotransmitter concentrations in the PVN

**Figure 3:**
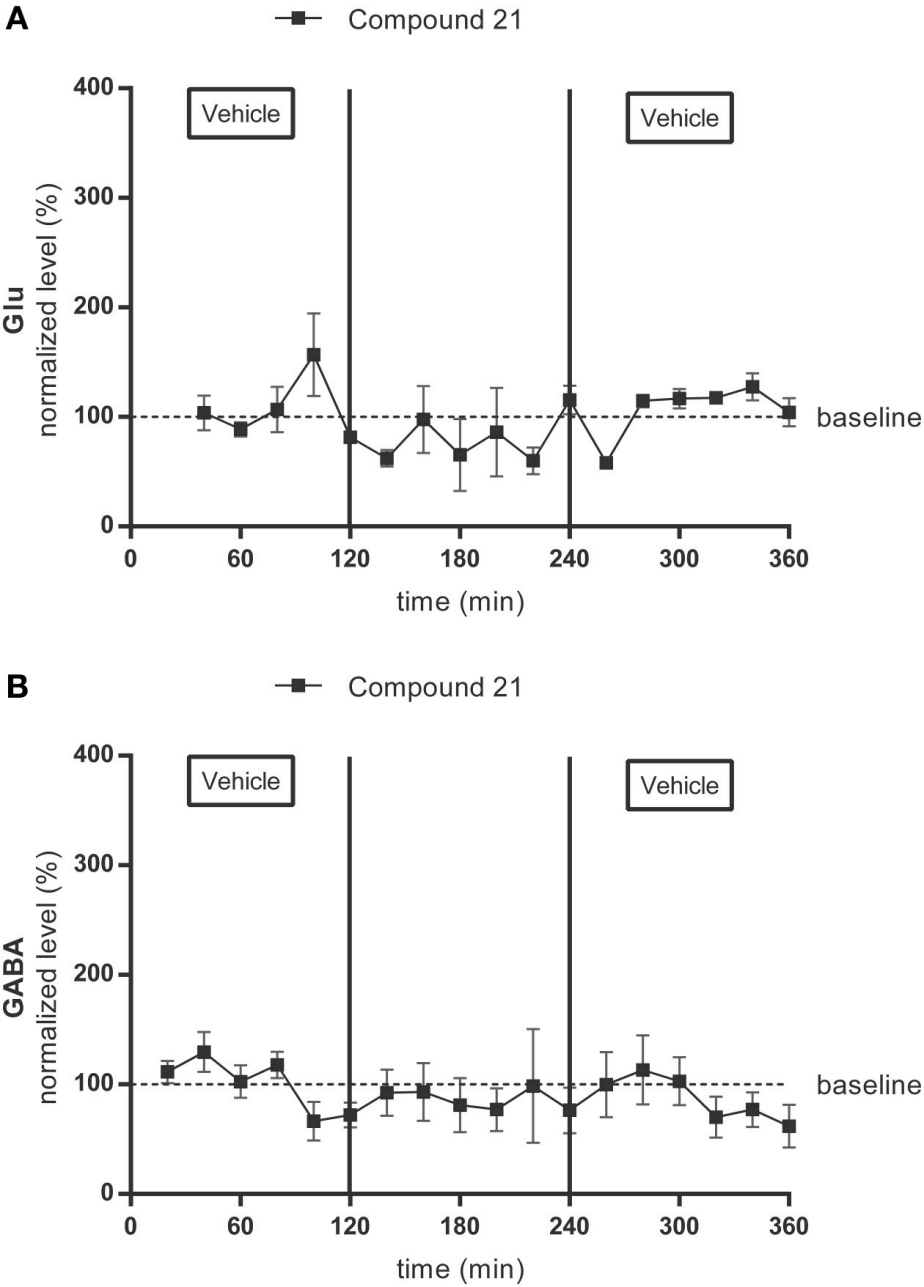
Microdialysis experiment: effect of infusion with the AT2R agonist C21 (0.05 μg/μl/h) on the extracellular glutamate (Glu) **(A)** (*n* = 4) and GABA **(B)** (*n* = 4) concentrations in the PVN in normotensive freely moving Wistar rats. Dialysates were collected every 20 min. C21 was dissolved in modified Ringer's solution and administered through the dialysis probe from time 120 to 240 min. Data are presented as the mean percentage of the baseline values (vehicle) ± SEM. Statistical analysis for intragroup differences of the treatment is performed using one-way ANOVA for repeated measures and the Dunnett's multiple comparison test.

Average baseline extracellular concentrations in the microdialysis samples of the PVN were 198 ± 107 nM for glutamate (Figure 3A) (*n* = 4) and 5 ± 4 nM for GABA (Figure 3B) (*n* = 4). Glutamate and GABA levels were not significantly altered by local infusion of C21 (0.05 μg/μl/h) (Figures 3A,B).

### AT2R-mediated MAP response to C21 infusion into the RVLM

**Figure 4:**
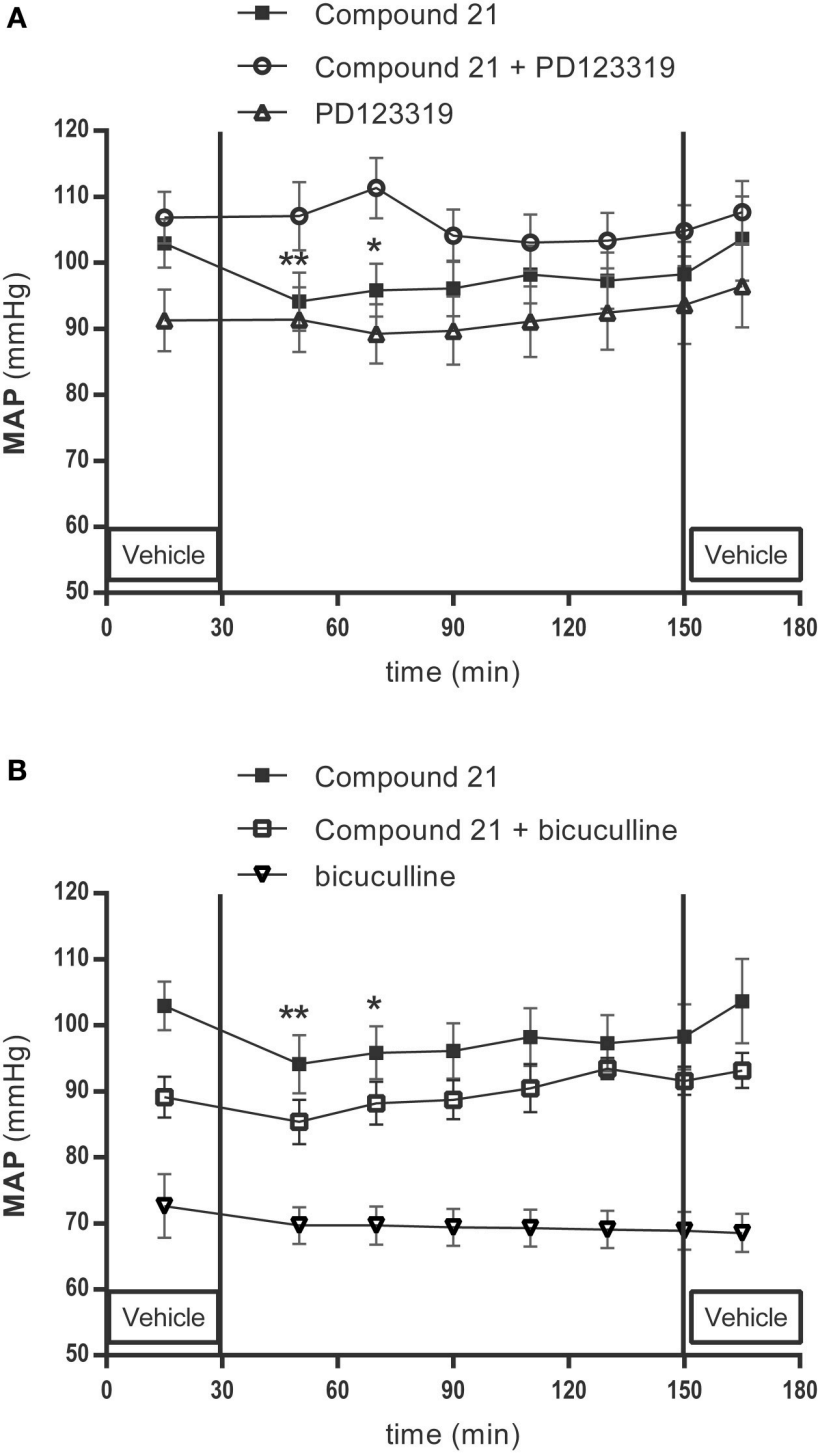
Mean arterial pressure (MAP) measurement experiment: effect of infusion in the RVLM with the AT2R agonist C21 (0.05 μg/μl/h) (*n* = 6), co-infusion of C21 with the AT2R antagonist PD123319 (0.05 μg/μl/h) (*n* = 4) and infusion of PD123319 (0.05 μg/μl/h) alone (*n* = 9) **(A)**; C21 (0.05 μg/μl/h) infusion alone, co-infusion of C21 (0.05 μg/μl/h) with the GABA-A R antagonist bicuculline (100μM) (*n* = 7), or infusion of bicuculline alone (*n* = 4) **(B)** on MAP in normotensive anesthetized Wistar rats. MAP was recorded every 2 s. C21, PD123319 and bicuculline were dissolved in modified Ringer's solution and administered through the dialysis probe from time 30 to 150 min. Data are shown as mean ± SEM. Statistical analysis for intragroup differences of the treatment is performed using one-way ANOVA for repeated measures and the Dunnett's multiple comparison test; significant data compared to the mean of baseline values are indicated by asterisks (^*^p < 0.05; ^**^p < 0.01).

Baseline MAP in Wistar rats were 96 ± 10 mmHg for the group receiving 0.01 μg/μl/h C21 (data not shown), and 103 ± 8 mmHg for the group receiving 0.05 μg/μl/h C21 (Figure 4A) (*n* = 6). Infusion of low dose C21 (0.01 μg/μl/h) within the RVLM did not change MAP (data not shown). Infusion of C21 into the RVLM for 120 min at a dose of 0.05 μg/μl/h significantly lowered MAP (-7 mmHg compared to baseline after 20 min of C21 infusion, *p* < 0.01; −6 mmHg after 40 min, *p* < 0.05) (Figure 4A).

Local infusion of PD123319 alone (0.05 μg/μl/h) did not modify the baseline MAP (94 ± 15 mmHg; Figure 4A) (*n* = 9).

Baseline MAP in the group of rats receiving co-infusion of C21 with PD123319 were 107 ± 7 mmHg (Figure 4A) (*n* = 4). Co-infusion of the AT2R antagonist PD123319 with C21 (0.05 μg/μl/h) abolished the C21 evoked decrease in MAP (Figure 4A).

Baseline MAP in the group of rats receiving co-infusion of C21 (0.05 μg/μl/h) with bicuculline were 89 ± 9 mmHg (Figure 4B) (*n* = 7). Co-infusion of the GABA-A antagonist bicuculline with C21 (0.05 μg/μl/h) abolished the C21 evoked decrease in MAP (Figure 4B).

### Lack of blood pressure response to C21 infusion into the PVN

**Figure 5:**
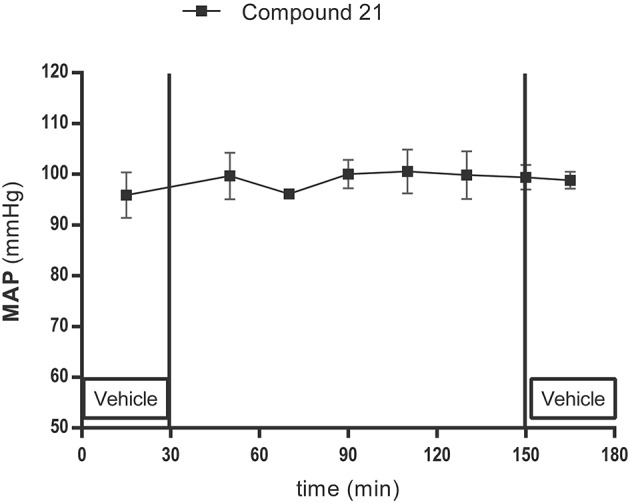
Mean arterial pressure (MAP) measurement experiment: lack of effect of AT2R stimulation and infusion with the AT2R agonist C21(0.05 μg/μl/h) in the PVN on MAP in normotensive anesthetized Wistar rats (*n* = 5). MAP was recorded every 2 s. C21 was dissolved in modified Ringer's solution and administered through the dialysis probe from time 30 to 150 min. Data are shown as mean ± SEM. Statistical analysis for intragroup differences of the treatment is performed using one-way ANOVA for repeated measures and the Dunnett's multiple comparison test.

Local infusion of C21 (0.05 μg/μl/h) into the PVN did not change baseline MAP (96 ± 8 mmHg; Figure 5; *n* = 5).

## Discussion

Although there is increasing evidence for a neuro- and cardioprotective role of the AT2R, several studies have indicated that stimulation of peripheral AT2R does not result in consistent blood pressure lowering effects (Yang et al., 2011; Steckelings et al., 2012; Brouwers et al., 2013; Matavelli and Siragy, 2015; Sumners et al., 2015). However, we recently demonstrated that icv infusion of the selective AT2R agonist C21 evoked a sustained hypotensive response in both normotensive and hypertensive rats, and that this central AT2R mediated hypotensive response is associated with sympatho-inhibition and increased baroreflex sensitivity (Brouwers et al., 2015), confirming and extending earlier results with icv administration of C21 in conscious normotensive Sprague-Dawley rats (Gao et al., 2011). Similar infusions in rats with heart failure also suppress sympathetic outflow by improving baroreflex sensitivity (Gao et al., 2014).

The major novel finding of the present study in male normotensive rats is that the hypotensive response to central administration of the selective non-peptide AT2R agonist C21 appears to be mediated at least in part by stimulation of AT2R located in the RVLM, whereas putative AT2R stimulation in the PVN does not seem to be involved. Indeed, local administration of C21 via microdialysis into the PVN did not alter local extracellular fluid neurotransmitter concentrations and did not reduce blood pressure. However, microdialysis administration of C21 into the RVLM resulted in a consistent blood pressure lowering effect and a significant increase in local GABA concentrations, and tended to decrease local glutamate concentrations. Moreover, these responses to local administration of C21 into the RVLM were abolished by local co-infusion with the selective AT2R antagonist PD123319 confirming that these responses are AT2R-mediated. There was some variation in baseline mean blood pressure between different groups of rats, which is not unusual in anesthetized rats from different batches. However, the reduction in mean blood pressure was very consistent and occurred in all animals at the same moment after starting the infusion of C21, with a return to baseline blood pressure levels after stopping the infusion. The absence of this effect of C21 in animals co-infused with either PD123319 or bicuculline was also consistent in all animals.

It is of interest to note that, whereas the effects of local administration of C21 were abolished by PD123319, indicating that exogenous stimulation of AT2R in the RVLM in normotensive rats results in a hypotensive response, local administration of PD123319 alone had no effect on blood pressure nor on neurotransmitter levels, suggesting that endogenous activation of AT2R in the RVLM is not involved in the regulation of blood pressure under basal conditions. This in line with our previously reported observations after icv infusion of C21 (Brouwers et al., 2015), and with those of Dai et al. (2015, 2016) who also reported that icv infusion of the AT2R antagonist had no effect on basal blood pressure. These authors further suggested that endogenous AT2R activation in the brain protects against the development of DOCA/salt induced hypertension in female, but not in male rats (Dai et al., 2015, 2016).

Most studies on central regulation of blood pressure target the RVLM, the PVN of the hypothalamus and the nucleus tractus solitarii (NTS) (Dampney et al., 2003; Guyenet, 2006; Dupont and Brouwers, 2010). The RVLM receives mainly tonic excitatory signal projections from neurons in the PVN. The RVLM presympathetic neurons, the major source of sympatho-excitatory outflow from the brain, project to sympathetic preganglionic neurons in the spinal cord (Sun et al., 1988; Pan, 2004; Kantzides and Badoer, 2005; Dupont and Brouwers, 2010). The excitatory drive from the RVLM originates from glutamatergic neurons (Ross et al., 1984; Guyenet et al., 2004; Dupont and Brouwers, 2010), which are tonically active under resting conditions but can be modulated by both excitatory and inhibitory synaptic inputs (Dampney et al., 2003). The neuronal activity of the RVLM region is modulated indirectly by input from the NTS, where baroreceptor afferents terminate, by the PVN, and by the caudal ventrolateral medulla (CVLM), which has an inhibitory influence on RVLM neurons (Schreihofer and Guyenet, 2002; Dupont and Brouwers, 2010). Therefore, the RVLM region is considered the most important site in the central regulation of sympathetic tone and blood pressure (Pointer, 2005; Guyenet, 2006; Dupont and Brouwers, 2010).

Several studies have indicated that the hypertensive response and the increased sympathetic tone evoked by central Ang II administration involves the activation of AT1R on spinally projecting glutamatergic vasomotor neurons located in the RVLM, which then further directly or indirectly elevate the sympathetic outflow (Hu et al., 1985; Dupont and Brouwers, 2010).

Although initial studies using receptor binding techniques and autoradiography studies suggested that central nervous system (CNS) cardiovascular control areas in the brainstem such as the RVLM are devoid of or only express low levels of AT2R (Millan et al., 1991; Lenkei et al., 1997; Hu et al., 1985), observations made in earlier functional studies did support a role for AT2R within the RVLM. Gao et al suggested that AT2R in the RVLM exhibit an inhibitory effect on sympathetic outflow and suggested down-regulation of AT2R in the RVLM as a contributory factor in the sympatho-excitation in congestive heart failure (Gao et al., 2008b). The same group further reported that overexpression of AT2R within the RVLM in normotensive rats reduced blood pressure, probably by sympatho-inhibition (Gao et al., 2008a). Further, Tedesco and Ally reported that the pressor and tachycardic responses to static muscle contraction were enhanced by selective blockade of AT2R in the RVLM in anaesthetized rats (Tedesco and Ally, 2009). In addition, electrophysiological studies in AT1Ra knockout mice suggest that AT2R play an antagonistic role against AT1R mediated actions of Ang II through AT2R mediated hyperpolarization and decrease in firing rate in bulbospinal RVLM neurons (Matsuura et al., 2005). The results of the present study in male normotensive rats validate and extend the results of these earlier studies and support the hypothesis that functional AT2R are present within the RVLM of normotensive rats and that their selective stimulation mediates a blood pressure lowering response probably mediated by sympatho-inhibition.

The results are also in line with those of a recent study that used a reporter mouse strain to provide an in-depth analysis of cellular and regional localization of AT2R in the mouse brain (de Kloet et al., 2016b). These investigations showed that AT2R are present in or near different brain sites involved in blood pressure regulation. These authors did not observe AT2R positive neurons within the PVN, which is in line with our observation that local administration of C21 within the PVN had no effect on blood pressure and neurotransmitter levels, confirming the absence of functional AT2R within the PVN. However, they found indications that AT2R are localized on efferents terminating in the PVN and within GABAergic neurons surrounding this nucleus (de Kloet et al., 2016b). They further reported that patch-clamp electrophysiological experiments revealed that selective activation of AT2R not within the PVN but in the peri-PVN area using C21 facilitates inhibitory (i.e., GABAergic) neurotransmission and leads to reduced activity of arginine vasopressin neurons within the PVN (de Kloet et al., 2016a,b).

Although not many AT2R were observed on neuronal cell bodies within the RVLM, de Kloet et al reported that the RVLM (and also the CLVM) are densely populated with AT2R positive nerve terminals/fibers (de Kloet et al., 2016b). It is therefore possible that presynaptic AT2R are expressed on nerve terminals within the RVLM and that their activation may influence neurotransmitter release from these terminals.

Of particular interest is also the observation by de Kloet et al. that AT2R containing neurons in the hindbrain are primarily GABAergic (de Kloet et al., 2016b). This may be important as GABA is known to have potent inhibitory actions within the RVLM (Menezes and Fontes, 2007). Projections of inhibitory GABAergic neurons to the RVLM region decrease its output to the sympathetic preganglionic regions. The CVLM which receives input from the NTS that is stimulated following blood pressure elevation, is one of the important sources of GABA (Blessing and Li, 1989; Dampney et al., 2003; Dupont and Brouwers, 2010). It was previously shown that the tonic excitatory AT1R mediated effect of Ang II on RVLM sympatho-excitatory neurons in normotensive animals is unmasked when tonic inhibitory GABAergic output is blocked (Tagawa et al., 2000). These and other observations suggest that the overall AT1R mediated effect on sympathetic tone of brain Ang II may depend on a balance between the activation of excitatory glutamatergic neurons and the inhibitory GABAergic neurons, which are both known to express AT1R (Dupont and Brouwers, 2010). Pharmacological blockade of GABA-A receptors in the sympathoexcitatory region of the RVLM has previously been shown to almost entirely eliminate the action of caudal inhibitory vasomotor neurons resulting in increased sympathetic tone and blood pressure (Blessing and Li, 1989; Tagawa et al., 2000), whereas GABA-A receptor stimulation in the RVLM lowered blood pressure (Menezes and Fontes, 2007) indicating a functional sympatho-inhibitory role for GABA-A receptors within the RVLM, in contrast to GABA-B receptors, the stimulation of which does not reduce blood pressure (Menezes and Fontes, 2007).

It is therefore tempting to speculate that the hypotensive response to local administration of C21 within the RVLM observed in the present study might be mediated by stimulation of presynaptic AT2R located on inhibitory GABAergic nerve terminals resulting in increased GABA release and subsequent GABA-A receptor-mediated reduction in sympathetic tone. Our observations of a significant increase in local GABA concentration after C21 administration, which was also abolished by co-infusion of the AT2R antagonist, and that the hypotensive response was equally abolished by local administration of the GABA-A receptor antagonist bicuculline, are in line with this hypothesis.

In addition, nitric oxide may also be involved in the observed increase in GABA within the RVLM. Nitric oxide is indeed also an important mediator within the RVLM acting on presynaptic terminals to increase GABA release (Kishi et al., 2001; Shinohara et al., 2012) and impacts on central AT2R mediated modulation of baroreflex regulation (Abdulla and Johns, 2014). This is also in line with our previous observation that the hypotensive and sympatho-inhibitory response to chronic selective stimulation of central AT2R through chronic icv infusion of C21, required a functioning central nitric-oxide pathway (Brouwers et al., 2015).

As mentioned above, we and others found no evidence that endogenous activation of AT2R in the RVLM contributes significantly to the control of blood pressure under basal conditions. This could be due to the dominant Ang II dependent AT1R mediated balance between glutamatergic and GABAergic activity, and may be different in pathologic conditions associated with sympatho-excitation.

Nevertheless, if confirmed by further studies, the presence of AT2R on GABAergic sympatho-inhibitory nerve terminals within the RVLM could open the possibility to develop selective AT2R agonists as a possible new therapy for conditions characterized by increased sympathetic activity. C21 barely crosses the blood-brain-barrier (Shraim et al., 2011), therefore the development of more lipophilic AT2R agonists would be needed to target AT2R within the RVLM as a possible new antihypertensive strategy.

The present study has some limitations. The study was done in normotensive male rats only and some studies have indicated that the role of AT2R in regulating blood pressure may be sex specific (Hilliard et al., 2012; Dai et al., 2015, 2016). Moreover, different or more pronounced responses may be observed in future similar studies in rat models of hypertension.

We observed differences in baseline glutamate and GABA levels between different groups of rats. Such variations in baseline neurotransmitter values, are not unusual and also not a problem, taken into account that the conclusions we drew from our experiments are based on relative changes in transmitter levels in response to the interventions. Our group as well as others previously reported substantial intrastrain differences in for example hippocampal extracellular levels of noradrenalin, dopamine, serotonin, glutamate and GABA (Miller et al., 1968; Portelli et al., 2009). Therefore, we believe that observing intrastrain differences between different batches, even coming from the same vendor, in basal glutamate and GABA levels within the PVN and the RVLM is not unexpected. In addition all rats were outbred, and as described by Yilmazer-Hanke (2008), outbred strains are genetically heterogeneous populations with a high intrastrain variation. Another important factor that needs to be taken into account related to observed variations in our baseline measurements is the yield of a microdialysis probe. Microdialysis does not reflect the absolute values present in the extracellular environment. Indeed, due to the kinetic process of dialysis the yield is not 100%, which is not unusual. However, microdialysis is an excellent tool to measure relative changes in concentrations in function of time after an intervention. There may be variation in probe yields between different experiments, but for each animal the effect (relative increase in GABA) occurred always at the same time (shortly after the start of the C21 administration), and the levels returned again to baseline after withdrawal of the C21 infusion. This relative change from baseline in GABA levels in response to C21 was consistently observed during the same time period in each rat. Moreover, in each rat, GABA levels remained stable when co-infusion with the AT2R antagonist. Further, the results were confirmed when expressed as AUC of GABA values before-during- and after C21 infusion (Figure 2).

The fact that we did not observe an increase in blood pressure after bicuculline alone does not exclude a tonic inhibitory GABA output as previously suggested by Smith and Barron (1990). These authors reported an increase in blood pressure after bilateral microinjections of bicuculline. In the present study, we administered bicuculline unilaterally into the left-sided RVLM only, hence leaving the GABA receptors in the RVLM at the other side unaffected. We assume that a blood pressure increase resulting from interruption of a putative tonic inhibitory GABAergic tone can only be detected after bilateral GABA receptor blockade within the RVLM.

It would also be of interest to further explore the effect of local administration of C21 into the NTS which is also known to contain AT2R (de Kloet et al., 2016b).

In conclusion, the results of the present study provide evidence that acute stimulation of AT2R within the RVLM by the non-peptide AT2R agonist C21 lowers blood pressure and increases local GABA release in normotensive rats, and that this hypotensive response requires functional GABA-A receptors.

## Author contributions

LL, First author, performed the research and wrote the research paper; SB, Co-promoter and supervisor of practical work and design of the reseach study; IJS, Co-promotor and design of the research study and critical insights in the research paper (Head of Center for Neurosciences, VUB); AD, Promotor Design of the research study and critical insights in the research paper (Head of the department of Clinical Pharmacology and Clinical Pharmacy, UZ Brussel).

### Conflict of interest statement

The authors declare that the research was conducted in the absence of any commercial or financial relationships that could be construed as a potential conflict of interest.

## References

[B1] Abdulla M. H. Johns E. J. (2014). Nitric oxide impacts on angiotensin AT2 receptor modulation of high-pressure baroreflex control of renal sympathetic nerve activity in anaesthetized rats. Acta Physiol. 210, 832–844. 10.1111/apha.12207 24279649 PMC3992911

[B2] Blessing W. W. Li Y. W. (1989). Inhibitory vasomotor neurons in the caudal ventrolateral region of the medulla oblongata. Prog. Brain Res. 81, 83–97. 10.1016/S0079-6123(08)62000-2 2694225

[B3] Brouwers S. Smolders I. Massie A. Dupont A. G. (2013). Angiotensin II type 2 receptor-mediated and nitric oxide-dependent renal vasodilator response to compound 21 unmasked by angiotensin-converting enzyme inhibition in spontaneously hypertensive rats *in vivo*. Hypertension 62, 920–926. 10.1161/HYPERTENSIONAHA.112.00762 24041944

[B4] Brouwers S. Smolders I. Wainford R. D. Dupont A. G. (2015). Hypotensive and sympathoinhibitory responses to selective central AT2 receptor stimulation in spontaneously hypertensive rats. Clin. Sci. 129, 81–92. 10.1042/CS20140776 25655919 PMC4430196

[B5] Butcher K. S. Cechetto D. F. (1998). Receptors in lateral hypothalamic area involved in insular cortex sympathetic responses. Am. J. Physiol. 275(2 Pt 2), H689–H696. 9683459 10.1152/ajpheart.1998.275.2.H689

[B6] Dai S. Y. Peng W. Zhang Y. P. Li J. D. Shen Y. Sun X. F. (2015). Brain endogenous angiotensin II receptor type 2 (AT2-R) protects against DOCA/salt-induced hypertension in female rats. J. Neuroinflammation 12:47. 10.1186/s12974-015-0261-47 25885968 PMC4355980

[B7] Dai S. Y. Zhang Y. P. Peng W. Shen Y. He J. J. (2016). Central infusion of Angiotensin II Type 2 receptor agonist compound 21 attenuates DOCA/NaCl-induced hypertension in female rats. Oxid. Med. Cell. Longev. 2016:3981790. 10.1155/2016/3981790 26783414 PMC4691472

[B8] Dampney R. A. Horiuchi J. Tagawa T. Fontes M. A. Potts P. D. Polson J. W. (2003). Medullary and supramedullary mechanisms regulating sympathetic vasomotor tone. Acta Physiol. Scand. 177, 209–218. 10.1046/j.1365-201X.2003.01070.x 12608991

[B9] de Gasparo M. Catt K. J. Inagami T. Wright J. W. Unger T. (2000). International union of pharmacology. XXIII. The angiotensin II receptors. Pharmacol. Rev. 52, 415–472. 10977869

[B10] de Kloet A. D. Pitra S. Wang L. Hiller H. Pioquinto D. J. Smith J. A.. (2016a). Angiotensin Type-2 receptors influence the activity of vasopressin neurons in the paraventricular nucleus of the hypothalamus in male mice. Endocrinology 157, 3167–3180. 10.1210/en.2016-1131 27267713 PMC4967126

[B11] de Kloet A. D. Wang L. Ludin J. A. Smith J. A. Pioquinto D. J. Hiller H.. (2016b). Reporter mouse strain provides a novel look at angiotensin type-2 receptor distribution in the central nervous system. Brain Struct. Funct. 221, 891–912. 10.1007/s00429-014-0943-1 25427952 PMC4446257

[B12] Dupont A. G. Brouwers S. (2010). Brain angiotensin peptides regulate sympathetic tone and blood pressure. J. Hypertens. 28, 1599–1610. 10.1097/HJH.0b013e32833af3b2 20502352

[B13] Gao J. Zhang H. Le K. D. Chao J. Gao L. (2011). Activation of central angiotensin type 2 receptors suppresses norepinephrine excretion and blood pressure in conscious rats. Am. J. Hypertens. 24, 724–730. 10.1038/ajh.2011.33 21394088 PMC3286515

[B14] Gao J. Zucker I. H. Gao L. (2014). Activation of central angiotensin type 2 receptors by compound 21 improves arterial baroreflex sensitivity in rats with heart failure. Am. J. Hypertens. 27, 1248–1256. 10.1093/ajh/hpu044 24687998 PMC4229732

[B15] Gao L. Wang W. Li H. Sumners C. Zucker I. H. (2008a). Effects of angiotensin type 2 receptor overexpression in the rostral ventrolateral medulla on blood pressure and urine excretion in normal rats. Hypertension 51, 521–527. 10.1161/HYPERTENSIONAHA.107.101717 18086951

[B16] Gao L. Wang W. Z. Wang W. Zucker I. H. (2008b). Imbalance of angiotensin type 1 receptor and angiotensin II type 2 receptor in the rostral ventrolateral medulla: potential mechanism for sympathetic overactivity in heart failure. Hypertension 52, 708–714. 10.1161/HYPERTENSIONAHA.108.116228 18768398 PMC2760297

[B17] Gao L. Zucker I. H. (2011). AT2 receptor signaling and sympathetic regulation. Curr. Opin. Pharmacol. 11, 124–130. 10.1016/j.coph.2010.11.004 21159555 PMC3075409

[B18] Guyenet P. G. (2006). The sympathetic control of blood pressure. Nat. Rev. Neurosci. 7, 335–346. 10.1038/nrn1902 16760914

[B19] Guyenet P. G. Stornetta R. L. Weston M. C. McQuiston T. Simmons J. R. (2004). Detection of amino acid and peptide transmitters in physiologically identified brainstem cardiorespiratory neurons. Auton. Neurosci. 114, 1–10. 10.1016/j.autneu.2004.06.003 15331039

[B20] Hatam M. Ganjkhani M. (2012). Effect of GABA(A) receptors in the rostral ventrolateral medulla on cardiovascular response to the activation of the bed nucleus of the stria terminalis in female ovariectomized rats. Iran J. Med. Sci. 37, 242–252. 23390330 PMC3565197

[B21] Hilliard L. M. Jones E. S. Steckelings U. M. Unger T. Widdop R. E. Denton K. M. (2012). Sex-specific influence of angiotensin type 2 receptor stimulation on renal function: a novel therapeutic target for hypertension. Hypertension 59, 409–414. 10.1161/HYPERTENSIONAHA.111.184986 22158645

[B22] Hu L. Zhu D. N. Yu Z. Wang J. Q. Sun Z. J. Yao T. (1985). Expression of angiotensin II type 1 (AT(1)) receptor in the rostral ventrolateral medulla in rats. J. Appl. Physiol. 92, 2153–2161. 11960969 10.1152/japplphysiol.00261.2001

[B23] Kantzides A. Badoer E. (2005). nNOS-containing neurons in the hypothalamus and medulla project to the RVLM. Brain Res. 1037, 25–34. 10.1016/j.brainres.2004.11.032 15777749

[B24] Kishi T. Hirooka Y. Sakai K. Shigematsu H. Shimokawa H. Takeshita A. (2001). Overexpression of eNOS in the RVLM causes hypotension and bradycardia via GABA release. Hypertension 38, 896–901. 11641305

[B25] Lenkei Z. Palkovits M. Corvol P. Llorens-Cortès C. (1997). Expression of angiotensin type-1 (AT1) and type-2 (AT2) receptor mRNAs in the adult rat brain: a functional neuroanatomical review. Front. Neuroendocrinol. 18, 383–439. 10.1006/frne.1997.0155 9344632

[B26] Li Y. F. Jackson K. L. Stern J. E. Rabeler B. Patel K. P. (2006). Interaction between glutamate and GABA systems in the integration of sympathetic outflow by the paraventricular nucleus of the hypothalamus. Am. J. Physiol. Heart Circ Physiol. 291, H2847–H2856. 10.1152/ajpheart.00625.2005 16877560

[B27] Li Y. Li X. H. Yuan H. (2012). Angiotensin II type-2 receptor-specific effects on the cardiovascular system. Cardiovasc. Diagn. Ther. 2, 56–62. 10.3978/j.issn.2223-3652.2012.02.02 24282697 PMC3839167

[B28] Li Z. Iwai M. Wu L. Shiuchi T. Jinno T. Cui T. X.. (2003). Role of AT2 receptor in the brain in regulation of blood pressure and water intake. Am. J. Physiol. Heart Circ. Physiol. 284, H116–H121. 10.1152/ajpheart.00515.2002 12388241

[B29] Matavelli L. C. Siragy H. M. (2015). AT2 receptor activities and pathophysiological implications. J. Cardiovasc. Pharmacol. 65, 226–232. 10.1097/FJC.0000000000000208 25636068 PMC4355033

[B30] Matsuura T. Kumagai H. Onimaru H. Kawai A. Iigaya K. Onami T.. (2005). Electrophysiological properties of rostral ventrolateral medulla neurons in angiotensin II 1a receptor knockout mice. Hypertension 46, 349–354. 10.1161/01.HYP.0000173421.97463.ac 15998710

[B31] Menezes R. C. Fontes M. A. (2007). Cardiovascular effects produced by activation of GABA receptors in the rostral ventrolateral medulla of conscious rats. Neuroscience 144, 336–343. 10.1016/j.neuroscience.2006.08.062 17049168

[B32] Millan M. A. Jacobowitz D. M. Aguilera G. Catt K. J. (1991). Differential distribution of AT1 and AT2 angiotensin II receptor subtypes in the rat brain during development. Proc. Natl. Acad. Sci. U.S.A. 88, 11440–11444. 10.1073/pnas.88.24.11440 1763058 PMC53151

[B33] Miller F. P. Cox R. H. Maickel R. P. (1968). Intrastrain difference in serotonin and norepinephrine in discrete areas of rat brain. Science 162, 463–464. 10.1126/science.162.3852.463 5683055

[B34] Miyawaki T. Minson J. Arnolda L. Chalmers J. Llewellyn-Smith I. Pilowsky P. (1996). Role of excitatory amino acid receptors in cardiorespiratory coupling in ventrolateral medulla. Am. J. Physiol. 271(5 Pt 2), R1221–R1230. 8945957 10.1152/ajpregu.1996.271.5.R1221

[B35] Namsolleck P. Recarti C. Foulquier S. Steckelings U. M. Unger T. (2014). AT(2) receptor and tissue injury: therapeutic implications. Curr. Hypertens. Rep. 16:416. 10.1007/s11906-013-0416-6 24414230 PMC3906548

[B36] Padia S. H. Carey R. M. (2013). AT2 receptors: beneficial counter-regulatory role in cardiovascular and renal function. Pflugers Arch. 465, 99–110. 10.1007/s00424-012-1146-3 22949090 PMC3548020

[B37] Pan H. L. (2004). Brain angiotensin II and synaptic transmission. Neuroscientist 10, 422–431. 10.1177/1073858404264678 15359009

[B38] Paxinos G. Watson S. (1998). The Rat Brain in Stereotaxic Coordinates, 2nd Edn. San Diego, CA: Academic Press.

[B39] Pointer M. A. (2005). Is central nitric oxide essential in hypertension? J. Hypertens. 23, 1637–1638. 10.1097/01.hjh.0000180352.99042.f3 16093906

[B40] Portelli J. Aourz N. De Bundel D. Meurs A. Smolders I. Michotte Y.. (2009). Intrastrain differences in seizure susceptibility, pharmacological response and basal neurochemistry of Wistar rats. Epilepsy Res. 87, 234–246. 10.1016/j.eplepsyres.2009.09.009 19833479

[B41] Ross C. A. Ruggiero D. A. Park D. H. Joh T. H. Sved A. F. Fernandez-Pardal J.. (1984). Tonic vasomotor control by the rostral ventrolateral medulla: effect of electrical or chemical stimulation of the area containing C1 adrenaline neurons on arterial pressure, heart rate, and plasma catecholamines and vasopressin. J. Neurosci. 4, 474–494. 6699683 10.1523/JNEUROSCI.04-02-00474.1984 PMC6564896

[B42] Schreihofer A. M. Guyenet P. G. (2002). The baroreflex and beyond: control of sympathetic vasomotor tone by GABAergic neurons in the ventrolateral medulla. Clin. Exp. Pharmacol. Physiol. 29, 514–521. 10.1046/j.1440-1681.2002.03665.x 12010201

[B43] Shinohara K. Hirooka Y. Kishi T. Sunagawa K. (2012). Reduction of nitric oxide-mediated gamma-amino butyric acid release in rostral ventrolateral medulla is involved in superoxide-induced sympathoexcitation of hypertensive rats. Circ. J. 76, 2814–2821. 10.1253/circj.CJ-12-0399 22972304

[B44] Shraim N. Mertens B. Clinckers R. Sarre S. Michotte Y. Van Eeckhaut A. (2011). Microbore liquid chromatography with UV detection to study the *in vivo* passage of compound 21, a non-peptidergic AT_2_, receptor agonist, to the striatum in rats. J. Neurosci. Methods 202, 137–142. 10.1016/j.jneumeth.2011.06.009 21723321

[B45] Siragy H. M. Inagami T. Ichiki T. Carey R. M. (1999). Sustained hypersensitivity to angiotensin II and its mechanism in mice lacking the subtype-2 (AT2) angiotensin receptor. Proc. Natl. Acad. Sci. U.S.A. 96, 6506–6510. 10.1073/pnas.96.11.6506 10339618 PMC26912

[B46] Smith J. K. Barron K. W. (1990). GABAergic responses in ventrolateral medulla in spontaneously hypertensive rats. Am. J. Physiol. 258(2 Pt 2), R450–R456. 1968724 10.1152/ajpregu.1990.258.2.R450

[B47] Smolders I. De Klippel N. Sarre S. Ebinger G. Michotte Y. (1995a). Tonic GABA-ergic modulation of striatal dopamine release studied by *in vivo* microdialysis in the freely moving rat. Eur. J. Pharmacol. 284, 83–91. 10.1016/0014-2999(95)00369-V 8549640

[B48] Smolders I. Sarre S. Michotte Y. Ebinger G. (1995b). The analysis of excitatory, inhibitory and other amino acids in rat brain microdialysates using microbore liquid chromatography. J. Neurosci. Methods 57, 47–53. 10.1016/0165-0270(94)00124-Y 7791364

[B49] Steckelings U. M. Larhed M. Hallberg A. Widdop R. E. Jones E. S. Wallinder C.. (2011). Non-peptide AT2-receptor agonists. Curr. Opin. Pharmacol. 11, 187–192. 10.1016/j.coph.2010.11.002 21167778

[B50] Steckelings U. M. Paulis L. Namsolleck P. Unger T. (2012). AT2 receptor agonists: hypertension and beyond. Curr. Opin. Nephrol. Hypertens. 21, 142–146. 10.1097/MNH.0b013e328350261b 22257799

[B51] Sumners C. de Kloet A. D. Krause E. G. Unger T. Steckelings U. M. (2015). Angiotensin type 2 receptors: blood pressure regulation and end organ damage. Curr. Opin. Pharmacol. 21, 115–121. 10.1016/j.coph.2015.01.004 25677800 PMC4380821

[B52] Sun M. K. Hackett J. T. Guyenet P. G. (1988). Sympathoexcitatory neurons of rostral ventrolateral medulla exhibit pacemaker properties in the presence of a glutamate-receptor antagonist. Brain Res. 438, 23–40. 10.1016/0006-8993(88)91320-0 2830940

[B53] Tagawa T. Fontes M. A. Potts P. D. Allen A. M. Dampney R. A. (2000). The physiological role of AT1 receptors in the ventrolateral medulla. Braz. J. Med. Biol. Res. 33, 643–652. 10.1590/S0100-879X2000000600005 10829092

[B54] Tasker J. G. Boudaba C. Schrader L. A. (1998). Local glutamatergic and GABAergic synaptic circuits and metabotropic glutamate receptors in the hypothalamic paraventricular and supraoptic nuclei. Adv. Exp. Med. Biol. 449, 117–121. 10.1007/978-1-4615-4871-3_11 10026791

[B55] Tedesco A. Ally A. (2009). Angiotensin II type-2 (AT2) receptor antagonism alters cardiovascular responses to static exercise and simultaneously changes glutamate/GABA levels within the ventrolateral medulla. Neurosci. Res. 64, 372–379. 10.1016/j.neures.2009.04.008 19379780

[B56] Van Hemelrijck A. Sarre S. Smolders I. Michotte Y. (2005). Determination of amino acids associated with cerebral ischaemia in rat brain microdialysates using narrowbore liquid chromatography and fluorescence detection. J. Neurosci. Methods 144, 63–71. 10.1016/j.jneumeth.2004.10.013 15848240

[B57] Wan Y. Wallinder C. Plouffe B. Beaudry H. Mahalingam A. K. Wu X.. (2004). Design, synthesis, and biological evaluation of the first selective nonpeptide AT2 receptor agonist. J. Med. Chem. 47, 5995–6008. 10.1021/jm049715t 15537354

[B58] Yang R. Smolders I. Vanderheyden P. Demaegdt H. Van Eeckhaut A. Vauquelin G.. (2011). Pressor and renal hemodynamic effects of the novel angiotensin A peptide are angiotensin II type 1A receptor dependent. Hypertension 57, 956–964. 10.1161/HYPERTENSIONAHA.110.161836 21464395

[B59] Yilmazer-Hanke D. M. (2008). Morphological correlates of emotional and cognitive behaviour: insights from studies on inbred and outbred rodent strains and their crosses. Behav. Pharmacol. 19, 403–434. 10.1097/FBP.0b013e32830dc0de 18690101

